# Effects of Sulfate Attack and Freeze–Thaw Cycles on Concrete with Compositely Modified Recycled Brick–Concrete Aggregate

**DOI:** 10.3390/ma19040698

**Published:** 2026-02-12

**Authors:** Ziming He, Mingyang Li, Jie Zhang, Aiqin Shen

**Affiliations:** 1 School of Civil and Architectural Engineering, Liaoning University of Technology, Jinzhou 121001, China; 2 Key Laboratory for Special Area Highway Engineering of Ministry of Education, Chang’an University, Xi’an 710064, China

**Keywords:** recycled brick–concrete aggregate, composite modification, recycled concrete, sulfate attack, freeze–thaw cycles, microstructure

## Abstract

In China, a significant portion of construction and demolition waste (CDW) consists of clay bricks and concrete, which can be processed into recycled brick–concrete aggregate (RBCA). This study explores the utilization of compositely modified RBCA as a substitute for natural coarse aggregate in concrete. Two distinct composite modification methods were applied to pretreat RBCA, and then the resistance of the resulting recycled brick–concrete aggregate concrete (RBCAC) to sulfate attack and freeze–thaw cycles was systematically examined and elucidated the underlying enhancement mechanisms. The experimental data revealed a clear trend: increasing the proportion of RBCA in the concrete mix correlates with a marked decline in its durability performance. In contrast, the application of composite modification techniques yielded a significant enhancement in durability. This improvement is primarily attributed to the mitigation of weak interfacial zones and the promotion of a more compact microstructure within the interfacial transition zone (ITZ). Consequently, the observed enhancement in durability metrics can be principally ascribed to this microstructural optimization. This research offers substantive theoretical insights that can facilitate the broader adoption of compositely modified RBCA in the production of sustainable concrete, contributing to waste valorization and resource conservation.

## 1. Introduction

China’s ongoing infrastructure development, integral to its national strategy of strengthening transportation networks, continues to generate substantial demand for building materials. Concurrently, the accumulation and disposal of construction and demolition waste (CDW) have risen correspondingly, with existing stockpiles estimated in the tens of billions of tons, creating significant environmental pressures [1,2]. Viewed through the lens of a circular economy, this waste stream holds significant potential as a source of recycled aggregates, which can be produced via standard processing techniques like crushing, magnetic separation, and screening [3,4]. Utilizing these recycled aggregates in construction projects offers a dual benefit: it mitigates the pressure on natural aggregate resources while simultaneously addressing the issues of land footprint and pollution stemming from waste disposal. Given the difficulty in separating CDW into single-type recycled aggregates, research on RBCA and its use in producing recycled concrete carries substantial practical significance. However, the widespread application of RBCA is hindered by its inherent technical drawbacks, such as inferior strength, high porosity, and a pronounced tendency for water absorption. These material deficiencies currently confine its use predominantly to non-structural applications like roadbeds and semi-rigid bases, thereby limiting its overall resource utilization efficiency [5,6].

In recent years, researchers have actively conducted scientific investigations into the application of recycled aggregates. Mefteh et al. [7] found that the water absorption process of recycled concrete aggregate is extremely rapid in the initial minutes after contact with water, which directly leads to a significant loss of slump in fresh concrete, thus necessitating effective control of their moisture condition to maintain the required workability. It is noteworthy that when using recycled concrete aggregate in the oven-dried state, additional water must be added to compensate for their high water absorption. However, this extra water is not fully absorbed by the recycled concrete aggregate during the short mixing period, resulting in its retention within the cement paste, ultimately leading to an increase in slump [8]. Therefore, in the actual mix design of recycled aggregate concrete, the amount of this additional water must be strictly controlled to precisely regulate the workability of fresh concrete.

The aforementioned study primarily focuses on recycled concrete aggregate. However, when recycled aggregate contains brick components, the mechanisms through which they influence the properties of recycled concrete become considerably more complex. Hou et al. [9] revealed the effects of moisture condition and brick content in RBCA on the rheological properties of fresh recycled concrete. They established relationships between rheological parameters, thereby providing a reference for further understanding the properties of fresh RBCAC. According to orthogonal experimental results, Huang et al. [10] concluded that the ratio of recycled brick aggregate to recycled concrete aggregate had the most significant impact on the compressive and splitting tensile strength of RBCAC, followed by the dosage of water reducer and fiber content. Similarly, Hameed et al. [11] also found that the proportion of recycled brick aggregate in the coarse aggregate is the most critical factor affecting the strength of recycled aggregate concrete, with a higher proportion resulting in lower strength of the concrete. Through experimental investigations, Zhu et al. [12] obtained the full stress–strain curves of RBCAC under uniaxial compression, revealing that its brittleness index is significantly higher than that of ordinary concrete. This is primarily attributed to the presence of numerous initial defects within RBCA, which renders recycled concrete prepared with RBCA more prone to the development of extensive microcracks, thereby creating weak zones in the matrix [13].

Although the addition of mineral admixtures [14,15], optimizing mixing procedures [16,17], and mix proportion design can enhance the performance of recycled concrete to some extent [18,19], none of these methods fundamentally address the inherent drawbacks in recycled concrete caused by recycled aggregate. Previous studies have demonstrated that treatments such as mechanical grinding, acid solution immersion, polymer emulsion immersion, and pozzolanic slurry immersion can effectively enhance the properties of recycled aggregate, thereby improving the performance of recycled concrete prepared with treated recycled aggregate [20,21,22,23]. However, current research on the quality improvement of recycled aggregate mainly focuses on recycled concrete aggregate, and its findings may lack universality for RBCA. In addition, existing studies primarily investigate the preparation and mechanical properties of RBCAC, with few reports on its durability and microstructure.

In this study, two composite modification methods based on synergy theory were employed to pretreat RBCA: the CSS method (pozzolanic slurry coating-sodium silicate activation composite reinforcement) and the CSA method (pozzolanic slurry coating-silane coupling agent film-forming composite reinforcement). Subsequently, recycled concrete was prepared using the modified RBCA, and systematic durability tests were conducted under sulfate attack and freeze–thaw cycles. A corresponding damage prediction model was developed to achieve a quantitative assessment of the durability performance. Finally, through microstructural analysis, the distribution characteristics of hydration products and the evolution of pore structure in the ITZ were revealed, clarifying the enhancement mechanism of the composite modification strategy on the performance of recycled concrete. This research provides a theoretical basis and technical reference for the systematic improvement of the durability of RBCAC.

## 2. Materials and Methods

### 2.1. Materials

**Figure 1:**
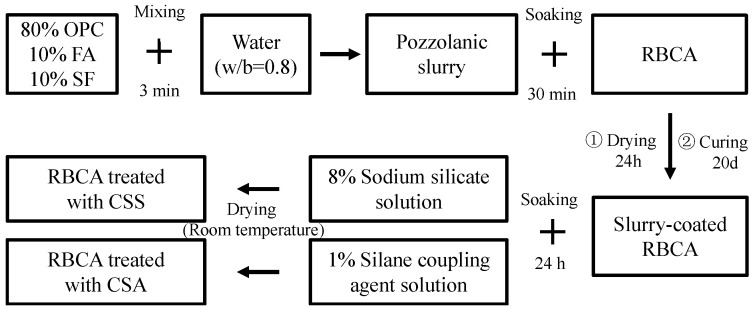
Schematic of the two composite modification treatment processes for RBCA.

The concrete formulations utilized ordinary Portland cement (42.5 R grade) and fly ash as the primary binders with specific surface areas of 0.3 m^2^/g and 0.2 m^2^/g, respectively. Fine aggregate was sourced from local river sand with a maximum particle size of 5 mm and a fineness modulus of 2.7. The coarse aggregates consisted of natural aggregate and recycled aggregate, with particle sizes ranging from 4.95 mm to 19 mm. The natural coarse aggregate (NCA) was granite, and the RBCA was obtained from a local recycling plant in Xi’an, China. Prior to use, the RBCA underwent a composite modification process following a previously established method [24]. The RBCA was initially washed with water to remove impurities and subsequently oven-dried to constant weight. It was then immersed in a blended strengthening slurry with a water-to-binder ratio of 0.8, in which 20% of the ordinary Portland cement (OPC) was replaced by fly ash (FA) and silica fume (SF). Following 30 min of immersion, the RBCA was taken out and allowed to air-dry for 24 h. It was then cured under standard conditions for 20 days. Subsequently, the slurry-coated RBCA was immersed in an 8% sodium silicate solution and a 1% silane coupling agent solution for 24 h. Finally, the treated RBCA was removed and air-dried for subsequent use. A schematic of the two composite modification treatment processes for RBCA is provided in Figure 1. The fundamental physical properties of all coarse aggregates are summarized in Table 1. To ensure adequate workability of the fresh concrete mixtures, a polycarboxylate-based high-range water reducer (SYW-3) was used at a dosage that achieved a water reduction rate of 27%. Mixing water was obtained from the laboratory’s tap supply.

### 2.2. Specimen Preparation

A standardized mixing procedure was consistently employed for all concrete formulations using a laboratory mixer. This study employed two concrete formulations with different water-to-binder ratios (*w*/*b*): a mix with *w*/*b* = 0.52 for C20 grade concrete and another with *w*/*b* = 0.45 for C30 grade concrete. Seven distinct mixtures were formulated by combining NCA with untreated or treated RBCA at replacement rates of 0%, 50%, and 100%. In this study, based on preliminary test results, the amount of additional water was determined as 75% of the 24 h water absorption of both untreated and treated RBCA. The concrete mix proportions used in this study are identical to those described in our previous work [25]. The specimen identification system begins with the concrete grade designation. The letter “R” is followed by the RBCA replacement rate, while the final letter combinations UT, CSS, and CSA correspond to untreated RBCA, slurry-coated RBCA combined with sodium silicate, and slurry-coated RBCA combined with silicon-based additive, respectively.

This study utilized 100 mm × 100 mm × 400 mm prismatic specimens to evaluate the concrete’s performance under sulfate attack and freeze–thaw cycles. The specimens were demolded one day after casting. This was followed by a 28-day curing period under stable laboratory conditions (20 ± 2 °C, >95% RH), ensuring they reached the required maturity before being subjected to the aggressive test environments.

### 2.3. Testing Methods

#### 2.3.1. Sulfate Attack Test

The sulfate attack test employed a wetting–drying cycle method, with each cycle consisting of 8 days of immersion in a 5% Na_2_SO_4_ solution followed by 7 days of air-drying at room temperature. This cycle design was developed with reference to the cycle method recommended in Chinese Standard GB/T 50082-2024 [26], and it incorporates the accelerated regimes commonly used in related studies on sulfate attack in concrete [27]. The aim was to balance the time required for sulfate ion penetration with the salt crystallization effect during the drying phase, thereby simulating the coupled chemical–physical damage under alternating wet and dry conditions in natural environments. To maintain the solution concentration, the sodium sulfate solution was replaced every 30 days. After each cycle, the mass and dynamic modulus of elasticity of the concrete specimens were measured. The weight loss rate and relative dynamic modulus of elasticity were calculated as follows:

(1) $$W_{n}=\frac{M_{0}-M_{n}}{M_{0}}\times 100\%$$

(2) $$E_{n}=\frac{{f_{n}}^{2}}{{f_{0}}^{2}}\times 100\%$$

The variables in the equations are defined as follows: *W_n_* represents the mass loss rate (%); *M*_0_ is the initial mass (g); *M_n_* represents the mass after *n* wetting–drying cycles (g); *E_n_* represents the relative dynamic elastic modulus (%); *f*_0_ represents the initial transverse fundamental frequency (Hz); and *f_n_* represents the transverse fundamental frequency after *n* wetting–drying cycles (Hz).

#### 2.3.2. Freeze–Thaw Test

The freeze–thaw test of the concrete was evaluated in accordance with the Chinese Standard GB/T 50082-2024, using a freeze–thaw test chamber (KDR-16, Beijing Zhongke Donghua Test Instrument Co., Ltd., Beijing, China). After the standard 28-day curing period, the specimens were immersed in water at 20 ± 2 °C for 4 days. They were then placed in the test chamber, with the water level maintained 20–30 mm above their tops. Each freeze–thaw cycle, which alternated between −20 °C and 20 °C, was completed within 2 to 4 h. After every 20 cycles, the specimens were removed, surface-dried, and measured for mass and dynamic elastic modulus. The mass loss rate and relative dynamic elastic modulus were calculated using Equations (1) and (2). In accordance with the standard, testing continues until a specimen’s relative dynamic elastic modulus falls below 60% or its mass loss reaches 5%, at which point the specimen is deemed to have failed and the experiment is concluded.

#### 2.3.3. Microstructural Characteristics

**Figure 2:**
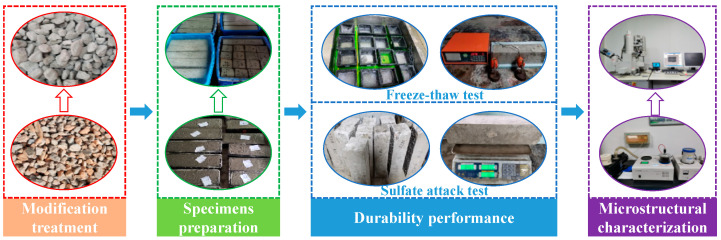
Experimental program of this study.

The microstructure of the ITZ in the concrete was observed using scanning electron microscopy (SEM, S-4800, Hitachi, Ltd., Tokyo, Japan). To prepare for observation, small specimens (approximately 10 mm cubes) were sectioned after removing the outer parts of the larger concrete cubes. The samples were immersed in absolute ethanol to stop hydration and then dried at 60 °C until a constant mass was achieved. Finally, the prepared specimens were sputter-coated with a thin layer of gold to enhance conductivity for SEM imaging. A schematic overview of the experimental program is provided in Figure 2.

## 3. Results and Discussion

### 3.1. Sulfate Attack Deterioration

#### 3.1.1. Mass Change

**Figure 3:**
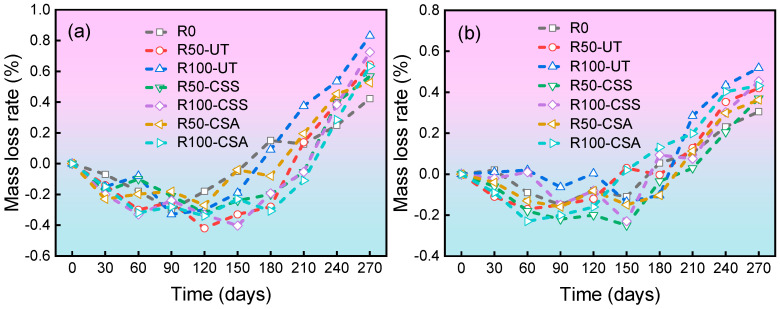
Mass loss rate versus time for concrete with different mix designs: (**a**) C20 grade; (**b**) C30 grade.

The mass loss rate results of concrete specimens after sulfate attack are shown in Figure 3. All mixtures initially exhibited a slight mass gain during the early testing stage, followed by a continuous decrease as the test progressed. Due to its relatively better compactness, the C30 concrete showed a less pronounced early-stage mass increase and a smaller mass loss during the later stages. In contrast, the C20 concrete, characterized by a higher water-to-binder ratio and a more porous structure, suffered significantly greater damage from sulfate attack. It was also observed that as the proportion of RBCA replacing NCA increased, the mass increase in the concrete specimens was more pronounced in the early stage of sulfate attack, and the time required to reach the peak mass was extended. A higher RBCA replacement ratio introduces greater internal defects within the concrete, thereby providing more space for the formation of expansive erosion products such as ettringite, calcium silicate, and gypsum. These expansive products will fill the pores within the concrete during their initial formation, resulting in a noticeable increase in the quality of the specimen and a temporary densification in the early stage of erosion [28,29].

After reaching the peak mass, the concrete specimens demonstrated an accelerated mass decline with increasing RBCA replacement ratios. The fundamental reason lies in the fact that when the expansive pressure generated by the reaction products exceeds the tensile strength of the concrete, it initiates the nucleation and propagation of microcracks. RBCA with more inherent defects further exacerbates this process, leading to the spalling of surface material, which manifests as accelerated mass loss. Additionally, the decalcification of C-S-H gel caused by the reaction between sulfate and cement hydration products weakens the cohesion of the matrix, collectively contributing to the mass loss. For C30 concrete, the mass loss rates of the R100-UT, R100-CSS, and R100-CSA groups after 270 days of sulfate exposure were 0.52%, 0.45%, and 0.43%, respectively. Furthermore, at a constant RBCA replacement rate, the concrete incorporating modified RBCA exhibited reduced mass loss, demonstrating that the treatment enhances the sulfate attack resistance of the RBCAC.

#### 3.1.2. Relative Dynamic Elastic Modulus

**Figure 4:**
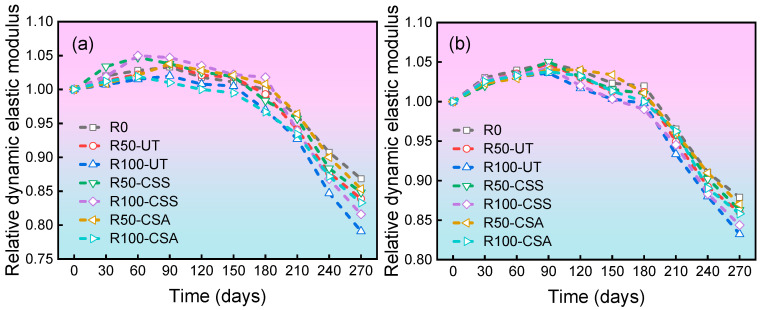
Relative dynamic elastic modulus versus time for concrete with different mix designs: (**a**) C20 grade; (**b**) C30 grade.

Figure 4 illustrates the variation in the relative dynamic elastic modulus of concrete subjected to different sulfate attack cycles, as evidenced by the data, which shows an initial slight increase followed by a distinct decrease. During the first 90 days of the sulfate attack test, the relative dynamic elastic modulus of all specimen groups remained above 1.00. This phenomenon is attributed to the dual mechanisms of ongoing cement hydration within the sulfate solution and the densification effect induced by the accumulation of corrosion byproducts.

For C30 concrete, the relative dynamic elastic modulus after 270 days of exposure were as follows: R0: 0.879; R50-UT: 0.860; R100-UT: 0.832; R100-CSS: 0.844; and R100-CSA: 0.858. The continuous decline in the dynamic elastic modulus of concrete marks the onset of structural damage. This is primarily driven by a coupled chemo-physical mechanism: (1) chemical expansion damage: the formation of ettringite and gypsum in the pore solution generates sustained expansive stress; (2) loss of cohesion: sulfate ions lead to the decalcification of the C-S-H gel [30]. A higher RBCA replacement rate increases the internal defects in the concrete, providing more pathways and space for ion migration and the accumulation of harmful products, thereby resulting in poorer sulfate attack resistance. The modification treatment significantly enhances matrix compactness by generating additional C-S-H gel and blocking pores. This densified microstructure effectively hinders the ingress rate of sulfate ions and suppresses the localized accumulation of expansive products in critical regions. Consequently, it alleviates internal stress concentration and the formation of microcracks, ultimately resulting in a slowed decline in the dynamic elastic modulus.

#### 3.1.3. Sulfate Attack Damage Model

Concrete, as a multiphase porous composite material, inevitably contains inherent microcracks within its internal structure. When subjected to sulfate attack, these microcracks propagate. Once the crack propagation reaches a critical level, it results in the deterioration of the concrete. Therefore, to quantify the relationships between the influencing factors and the evaluation indicators for the sulfate attack resistance of RBCAC, a multiple regression analysis was performed on the sulfate attack test results. This led to the establishment of a regression equation for the sulfate attack damage of RBCAC, thereby providing a reference and a theoretical basis for its durability design and assessment.

Based on the above results, the replacement rate of RBCA and the erosion duration are the main factors affecting the sulfate attack resistance of RBCAC. A linear relationship was observed between the RBCA replacement rate and both the mass loss rate and the relative dynamic elastic modulus, whereas a quadratic polynomial relationship was found between the erosion duration and these two parameters. Therefore, using the mass loss rate (*W_n_*) and relative dynamic elastic modulus (*E_n_*) of RBCAC as the dependent variables, and the RBCA replacement rate (*r*), the actual water-to-binder ratio (*w_s_*/*b*), and the erosion duration (*n*) as the independent variables, the regression equations for sulfate attack damage of RBCAC were established using Origin software (version 2022). The regression equations are presented in Equations (3) and (4), and the corresponding regression analysis results are summarized in Table 2.

(3) $$W_{n}=0.0342r-0.0877w_{s}/b+2.4901\times 10^{-5}n^{2}-0.0048n+0.0476$$

(4) $$E_{n}=-0.0013r-0.069w_{s}/b-5.6704\times 10^{-6}n^{2}+9.5567\times 10^{-4}n+1.0425$$

Table 2 shows that the F-values for both *W_n_* and *E_n_* of the two strength grades of RBCAC are greater than F_(α=0.05)_(5, 135) = 2.281. This indicates that the regression models are statistically significant, confirming a significant relationship between the independent variables (*r*, *w_s_*/*b*, *n*) and the dependent variables (*W_n_*, *E_n_*). Furthermore, the correlation coefficients for Equations (3) and (4) are 0.813 and 0.961, respectively, indicating that the models provide a good fit to the experimental data and have predictive capability for the sulfate attack resistance of RBCAC.

From Equation (3), it can be observed that the coefficient for the RBCA replacement rate *r* is positive, indicating that *W_n_* increases as *r* rises, reflecting the higher number of defects in RBCA, which exacerbates erosion damage. The coefficient for the *w_s_*/*b* is negative, suggesting that reducing the *w_s_*/*b* can decrease mass loss, primarily because it enhances the compactness of the RBCAC matrix. The first-order coefficient for erosion time *n* is negative, reflecting the temporary densification effect of early-stage erosion products filling the pores, while the second-order coefficient for erosion time *n* is positive, representing the accelerated phase of damage, which is mainly associated with the expansive damage caused by erosion products.

According to Equation (4), the coefficient for the RBCA replacement rate *r* is negative but has a small absolute value, indicating that its weakening effect on the dynamic elastic modulus is limited. This is primarily attributed to the enhanced interfacial strength resulting from the composite modification treatment. The coefficient for the *w_s_*/*b* is also negative, suggesting that reducing the *w_s_*/*b* can significantly increase the dynamic elastic modulus. This improvement is due to the increased density of the RBCAC matrix achieved by lowering the *w_s_*/*b*. The positive first-order coefficient for erosion time *n* reflects the stiffness enhancement effect on the material during the early stage of erosion due to the continued hydration of cement. During this stage, the formation of hydration products partially offsets the initial erosion damage. In contrast, the negative second-order coefficient for erosion time *n* reveals that, under prolonged sulfate attack, the dynamic elastic modulus exhibits an accelerated decline, which corresponds to the structural degradation stage characterized by accelerated microcrack propagation in the later phase of erosion.

It should be specifically noted that the predictive reliability of this model is established under specific material systems and environmental parameters. Currently, the model has been validated only for RBCAC with strength grades of C20 and C30. Its applicability to higher strength grades has not yet been verified and requires further calibration through targeted experiments. Additionally, if the modification process for the RBCA is altered, the variables in the model related to RBCAC performance must be recalibrated to ensure prediction accuracy.

### 3.2. Freeze–Thaw Deterioration

#### 3.2.1. Mass Change

**Figure 5:**
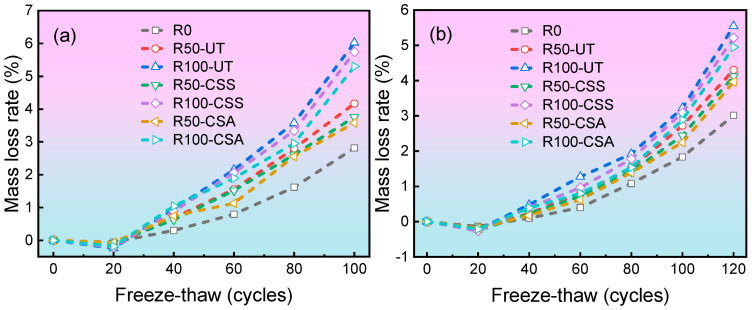
Mass loss rate versus freeze–thaw cycles for concrete with different mix designs: (**a**) C20 grade; (**b**) C30 grade.

Figure 5 presents the mass loss rate of concrete specimens subjected to different numbers of freeze–thaw cycles. It is evident that the mass loss rate of C20 concrete generally exceeds that of C30 concrete. This discrepancy arises from the more porous microstructure of lower-grade concrete, which can retain a greater volume of freeze-expandable water within its internal pores. Consequently, C20 concrete experiences more severe scaling damage under freeze–thaw cycles. The results also indicate that all concrete specimens exhibited an initial increase in mass during the early stages of freeze–thaw cycles. This phenomenon is attributed to the progressive expansion of microcracks within the concrete, which allows continuous absorption of external moisture into internal structure. Although surface scaling has already initiated at this stage, the mass of water absorbed exceeds the mass lost due to scaling, resulting in a net mass gain in the initial cycles. As the number of freeze–thaw cycles increases, the mass loss from scaling eventually surpasses the mass of absorbed water. This shift leads to a progressively increasing mass loss rate in subsequent cycles [31,32].

For the same number of freeze–thaw cycles, the concrete incorporating modified RBCA exhibited measurable reductions in mass loss rate compared to the control group. After 120 freeze–thaw cycles, the mass loss rates of the C30-R100-CSS and C30-R100-CSA groups were reduced by 6.0% and 10.7%, respectively, relative to the control group. This demonstrates that the modification treatment of RBCA can improve the frost resistance of the RBCAC. Moreover, the CSA modification method yielded a more pronounced improvement in the frost resistance of RBCAC than the CSS modification method.

#### 3.2.2. Relative Dynamic Elastic Modulus

**Figure 6:**
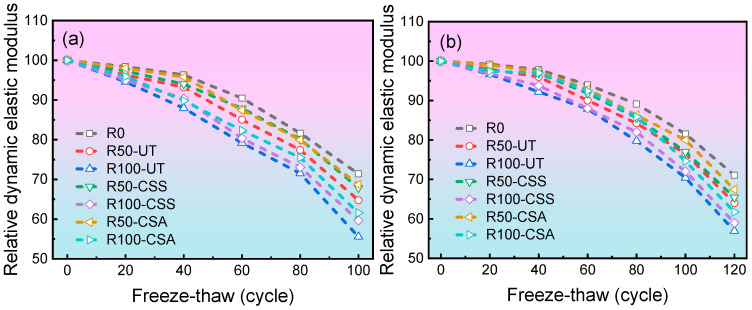
Relative dynamic elastic modulus versus freeze–thaw cycles for concrete with different mix designs: (**a**) C20 grade; (**b**) C30 grade.

Figure 6 illustrates the relative dynamic elastic modulus of concrete specimens after different numbers of freeze–thaw cycles. As shown in the figure, the relative dynamic elastic modulus of all concrete specimens exhibits a decreasing trend as the number of freeze–thaw cycles increases. For C30 concrete, after 120 freeze–thaw cycles, the relative dynamic elastic modulus of the R0, R50-UT, and R100-UT groups decreased to 71.1%, 64.0%, and 56.9%, respectively. Compared with the R100-UT group, the R100-CSS and R100-CSA groups demonstrated improvements of 3.62% and 8.47%, respectively. This enhancement can be attributed to the improved physical and mechanical properties of the RBCA after modification, which reduces the number of internal pores and microcracks in the RBCAC, thereby enhancing its frost resistance. Since the CSA modification yields more significant improvements in RBCA properties, the resulting RBCAC exhibits better frost resistance.

#### 3.2.3. Freeze–Thaw Damage Model

The Weibull distribution theory enables accurate large-sample estimation from limited datasets and has been widely applied in concrete engineering fields, particularly in reliability analysis and service life prediction [33,34]. Therefore, based on the experimental data from the freeze–thaw test, this study develops a damage progression model for RBCAC to predict its frost resistance service life.

Assuming that the time to failure *t* of RBCAC in a freeze–thaw environment follows a Weibull distribution, the probability density function *f*(*t*) of its freeze–thaw life can be expressed as follows:

(5) $$f(t)=\frac{\beta }{\eta }{\left(\frac{t}{\eta }\right)}^{\beta -1}\exp\left[-{\left(\frac{t}{\eta }\right)}^{\beta }\right]$$

The corresponding cumulative distribution function *F*(*t*) and reliability function *R*(*t*) are expressed as follows:

(6) $$F(t)=1-\exp\left[-{\left(\frac{t}{\eta }\right)}^{\beta }\right]$$

(7) $$R(t)=1-F(t)=\exp\left[-{\left(\frac{t}{\eta }\right)}^{\beta }\right]$$

where *η* is the scale parameter, *β* is the shape parameter, and *t* denotes the recorded value of the accelerated lifetime.

The freeze–thaw damage of concrete results from the progressive development of internal deterioration. Based on damage mechanics, the concrete damage index *D_t_* is introduced, expressed as follows:

(8) $$D_{t}=1-\frac{E_{t}}{E_{0}}$$

where *D_t_* represents the damage degree of the specimen after *t* freeze–thaw cycles; *E*_0_ denotes the initial dynamic elastic modulus of the specimen; and *E_t_* is the dynamic elastic modulus of the specimen after *t* freeze–thaw cycles.

**Figure 7:**
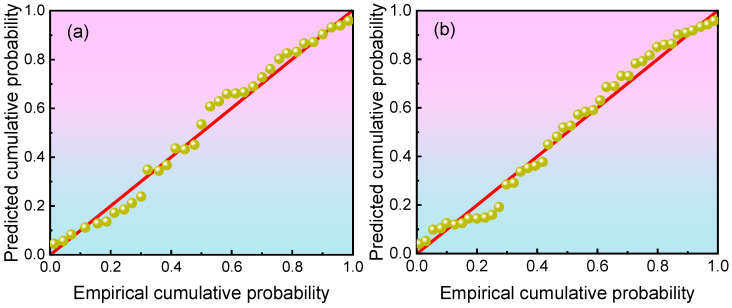
Results of the Weibull distribution goodness-of-fit test: (**a**) C20 grade; (**b**) C30 grade.

Using the damage degree data of two grades of concrete specimens during the freeze–thaw cycle process as sample information, a P-P plot was employed to test whether the damage degree data conforms to the Weibull distribution. The test results are shown in Figure 7. As can be seen from the figure, the data points in the P-P plot are evenly distributed on both sides of the *y* = *x* line, indicating that the damage degree data can be considered to follow the Weibull distribution.

Let *t*_1_ ≤ *t*_2_ ≤ … ≤ *t_n_* be a complete sample of size *n* from a Weibull distribution. Substituting them into Equation 6, and then rearranging the equation and taking double logarithms, the result is shown in the following equation.

(9) $$\ln t_{i}=\ln\eta +\frac{1}{\beta }\ln\left[\ln\frac{1}{1-F\left(t_{i}\right)}\right]\;,\;i=1,2,\ldots ,n$$

Let $x_{i}=\ln t_{i}$, $y_{i}=\ln\left[\ln\frac{1}{1-F\left(t_{i}\right)}\right]$, a=−β lnη and b=β, then Equation (9) can be rewritten as follows:

(10) $$y_{i}=a+bx_{i}$$

When *x*_i_ and *y*_i_ are known, the values of *a* and *b* can be obtained using the least squares method, as shown in the following equations:

(11) $$a=\frac{\sum {x_{i}}^{2}\sum y_{i}-\sum x_{i}\sum x_{i}y_{i}}{n\sum {x_{i}}^{2}-{\left(\sum x_{i}\right)}^{2}}$$

(12) $$b=\frac{n\sum x_{i}y_{i}-\sum x_{i}\sum y_{i}}{n\sum {x_{i}}^{2}-{\left(\sum x_{i}\right)}^{2}}$$

**Figure 8:**
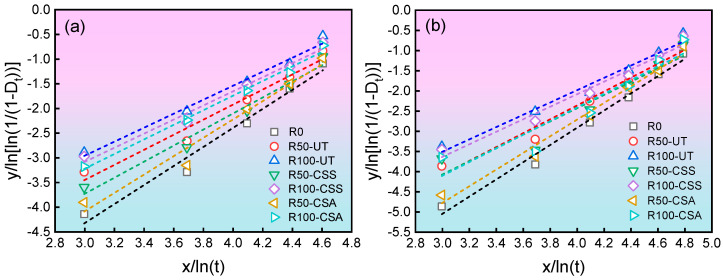
Linear fitting curves for concrete freeze–thaw damage data: (**a**) C20 grade; (**b**) C30 grade.

The aforementioned parameters were obtained by fitting using Origin software (version 2022), and the fitting results are shown in Figure 8. Based on the obtained intercept and slope, the shape parameter *β* and scale parameter *η* in the Weibull distribution model can be further determined. The corresponding parameter estimates are listed in Table 3.

**Figure 9:**
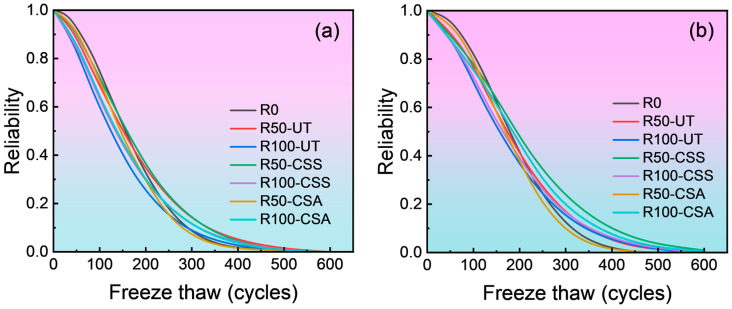
Reliability curve for freeze–thaw damaged concrete: (**a**) C20 grade; (**b**) C30 grade.

**Figure 10:**
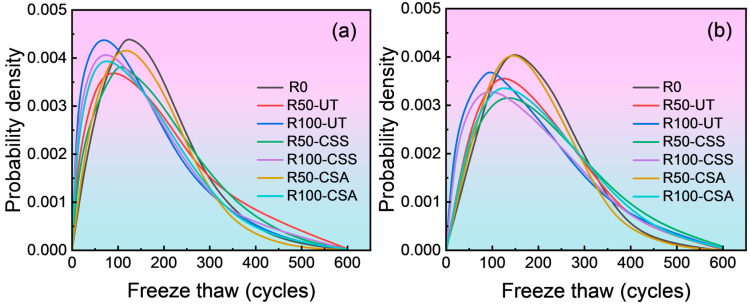
Probability density curve for freeze–thaw damaged concrete: (**a**) C20 grade; (**b**) C30 grade.

The values of *β* and *η* from Table 3 were substituted into Equations (5) and (7), respectively. The corresponding reliability curve and probability density curve were then plotted using Matlab software (version 2022), as shown in Figure 9 and Figure 10.

Analysis of Figure 9 reveals that the reliability of freeze–thaw damage for different types of concrete exhibits a three-stage variation pattern as the number of freeze–thaw cycles increases. These stages are characterized by a phase where reliability remains essentially at 1, a phase of rapid decline in reliability, and a final phase where reliability drops to 0. Moreover, as the number of freeze–thaw cycles increases, the reliability of the concrete specimens gradually decreases. According the relevant provisions of the “Standard for test methods of long-term performance and durability of ordinary concrete” (GB/T 50082-2024), the failure criterion for concrete is defined as when the relative dynamic elastic modulus drops to 60%. Therefore, when calculating the number of freeze–thaw cycles required for the relative dynamic elastic modulus to reach the failure threshold, the failure threshold *D_f_* is taken as 0.6.

Regarding C20 concrete, the numbers of freeze–thaw cycles to failure for the mixtures R0, R50-UT, R100-UT, R100-CSS and R100-CSA were 133, 124, 100, 109 and 113, respectively, and the corresponding values for C30 concrete were 158, 146, 128, 135 and 150. It can be concluded that concrete with a higher strength grade requires more freeze–thaw cycles to reach the same failure probability, indicating a slower rate of deterioration. This is attributed to the denser microstructure of higher-strength concrete, which retards the progression of freeze–thaw damage. Furthermore, the number of cycles endured by RBCAC before failure decreases as the RBCA content increases. However, subjecting the RBCA to a composite modification treatment can extend the failure time of RBCAC specimens. These findings are consistent with the laboratory test results, thereby validating the accuracy of the reliability function.

As observed in Figure 10, the probability density curves are approximately unimodal and symmetrical. Among them, the failure interval of the untreated RBCAC is positioned the furthest to the left, with a relatively small peak density value. This indicates that its reliability begins to decline first, but the rate of reliability decrease in the later stages is relatively slow. The composite treated RBCAC demonstrates intermediate values for both the failure interval and peak density, whereas ordinary concrete has the rightmost failure interval and the largest peak density, indicating the latest failure time but an accelerated rate of reliability loss once failure in initiated.

### 3.3. Microstructural Characterization

RBCAC is composed of recycled concrete aggregate (RCA), recycled brick aggregate (RBA), old mortar and new mortar. There are four types of ITZs contained in the RBCAC matrix, including the ITZ_1_ between RCA and new mortar, the ITZ_2_ between RCA and old mortar, the ITZ_3_ between old mortar and new mortar, and the ITZ_4_ between RBA and new mortar [35,36]. To better understand the strengthening mechanism of the properties of RBCAC by composite modification treatment, it is necessary to study the performance of multiple ITZs in RBCAC.

**Figure 11:**
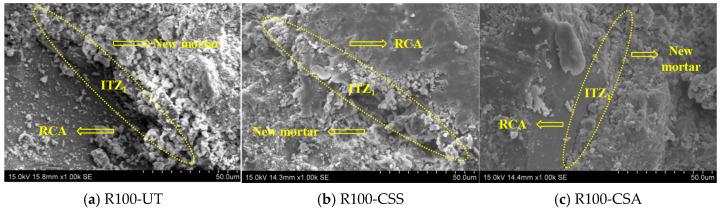
SEM images of ITZ_1_ in C30 concrete with unmodified and modified RBCA.

Figure 11 shows the SEM images of ITZ_1_ in RBCAC with the strength grade of 30 MPa. By comparing the SEM images of ITZ_1_ of the three groups of RBCAC, it can be seen that the RCA had a dense structure without obvious pores and microcracks. There were some irregular microcracks in the new mortar near ITZ_1_, and these microcracks were generated at the joint of ITZ_1_ and extended in the direction of the new mortar. Moreover, the hydration products near ITZ_1_ were mainly C-S-H gel and Ca(OH)_2_, accompanied by a small amount of ettringite. The pores of ITZ_1_ and the nearby mortar of R100-CSS and R100-CSA groups were less than those of the R100-UT group. These were mainly attributed to the following two aspects. On the one hand, the coating layer of pozzolan slurry on the RBCA surface continues to hydrate, and the generated hydration products gradually fill the pores and microcracks in ITZ_1_ and the nearby mortar matrix. On the other hand, the modification treatment reduces the water absorption of RBCA, which will reduce the water release of RBCA during the curing of the concrete specimens, thus improving the compactness of ITZ_1_ and the nearby mortar matrix.

**Figure 12:**
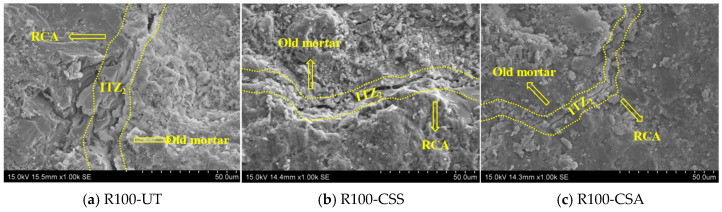
SEM images of ITZ_2_ in C30 concrete with unmodified and modified RBCA.

From the observation of Figure 12, it can be found that the content of hydration products in the mortar near ITZ_2_ was less. This is because ITZ_2_ originally existed in RCA and the waste concrete blocks used for many years have basically completed the hydration reaction [37], resulting in a significant reduction in the types and contents of hydration products of mortar near ITZ_2_ in RCA compared with ITZ_1_. In addition, the number of pores and microcracks near ITZ_2_ is influenced by the crushing process of waste concrete blocks. It can be observed that the modification treatment of RBCA can improve the microstructure near ITZ_2_ to a certain extent, narrowing the width of microcracks and increasing the number of hydration products.

**Figure 13:**
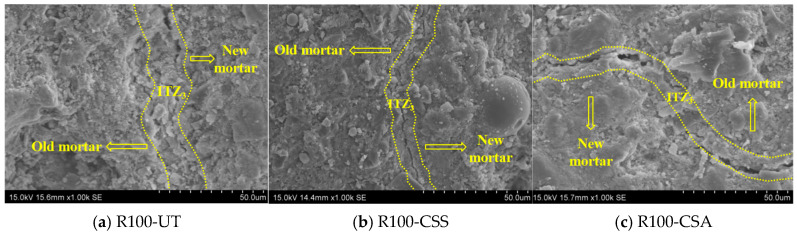
SEM images of ITZ_3_ in C30 concrete with unmodified and modified RBCA.

It can be seen from Figure 13 that there was no obvious difference between the new mortar and the old mortar on both sides of the ITZ_3_, which was due to the small difference in the elastic modulus of the two mortars, so they could be well combined together. Furthermore, the surface of the old mortar was rough, so it could mesh well with the new mortar, which helped to improve the properties of ITZ_3_ between the old mortar and new mortar. Modification treatment of RCA can improve the density of mortar on both sides of ITZ_3_. The reason is that the modified materials fills the pores in the old mortar on the surface of RCA. With the growth of age, the unhydrated particles in the old mortar and new mortar will continue to hydrate, and the generated hydration products will cross-grow and fill the pores and microcracks, thus making the microstructure near ITZ_3_ more dense.

**Figure 14:**
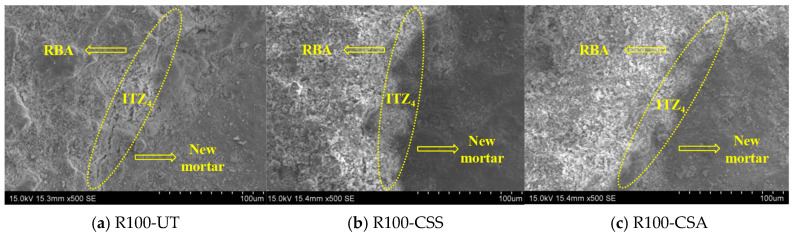
SEM images of ITZ_4_ in C30 concrete with unmodified and modified RBCA.

It can be found from Figure 14 that the microstructure of the ITZ_4_ between the RBA and new mortar was relatively dense, and the boundary was obvious. Through comparison, it can be observed that the ITZ_4_ between modified RBA and the new mortar was denser than that of the unmodified RBA. This is because the modified materials entering the pores and microcracks form a nested region with the RBA, thereby improving the performance of the RBA. In addition, in the curing process of concrete specimens, the hydration reaction will lead to the decrease in the relative humidity inside the concrete. At this time, the RBA will play the role of internal curing [38], which can promote the hydration reaction of the slurry near the ITZ_4_. The hydration products such as C-S-H gel and ettringite fill the pores and microcracks, presenting a dense microstructure of ITZ_4_.

## 4. Conclusions

In this paper, the durability and microstructure of recycled concrete made with unmodified and modified RBCA were studied. The conclusions could be drawn as follows:

(1) Under the action of sulfate attack, the mass change in RBCAC can be divided into three stages, which were the mass increase in the early stage of erosion, the slow decline in the middle stage of erosion and the rapid decline in the late stage of erosion. After the sulfate attack of 270 days, the mass loss rates of R100-UT, R100-CSS and R100-CSA groups in C30 concrete were 0.52%, 0.45% and 0.43%, and the relative dynamic elastic modulus were 0.832, 0.844 and 0.858, respectively. A regression equation for the sulfate attack damage of RBCAC was developed using multiple regression analysis.

(2) With the increase in RBCA replacement rates, the mass loss rate and relative dynamic elastic modulus loss rate of RBCAC increased. After 120 freeze–thaw cycles, the relative dynamic elastic modulus of R100-CSS and R100-CSA groups in C30 concrete increased by 3.62% and 8.47%, respectively, compared with that of R100-UT group.

(3) The damage degree (*D_f_*) was introduced as a freeze–thaw damage indicator for concrete specimens. The parameters of the Weibull distribution were estimated using linear regression, and the established model accurately described the entire degradation process of the freeze–thaw cycles of RBCAC. When the reliability was 0.6, the numbers of freeze–thaw cycles to failure for the R0, R50-UT, R100-UT, R100-CSS and R100-CSA groups in C30 concrete were 158, 146, 128, 135, and 150, respectively.

(4) There are four types of ITZs in the RBCAC matrix. A significant amount of C-S-H gel and Ca(OH)_2_, along with a small quantity of ettringite crystals, are present at ITZ_1_. The hydration product content in ITZ_2_ and the nearby mortar is relatively low but exhibits stable performance. The difference between the new and old mortars on either side of ITZ_3_ is minimal. A distinct boundary is observed between the RBA and new mortar at ITZ_4_. Composite modification treatment can reduce the width of the ITZs and increase their compactness, which is primarily attributed to the enhanced strength and reduced water absorption of RBCA after composite modification treatment.

## Figures and Tables

**Table 1:** Fundamental physical properties of the coarse aggregates.

Material	Apparent Density (kg/m^3^)	Water Absorption (%)	Crushing Value (%)
NCA	2704	0.5	10.6
RBCA	2552	11.8	22.8
RBCA_CSS	2598	11.0	17.6
RBCA_CSA	2462	8.0	17.7

**Table 2:** Regression analysis results.

Indicator	Source	Sum of Squares	Degree of Freedom	Mean Square	*F* Value	*R*-Squared
*W_n_*	Regression	7.694	5	1.539	121.904	0.813
	Residual	1.704	135	0.013		
	Total	9.398	140			
*E_n_*	Regression	135.309	5	27.062	169,508.669	0.961
	Residual	0.022	135	1.596 × 10^−4^		
	Total	135.331	140			

**Table 3:** Summary of fitted parameter estimates for concrete freeze–thaw damage.

Mix ID	Parameters				
	*a*	*b*	*R^2^*	*β*	*η*
C20-R0	−10.1017	1.9273	0.9669	1.9273	188.9295
C20-R50-UT	−8.0221	1.5252	0.9568	1.5252	192.4245
C20-R100-UT	−7.2046	1.4165	0.9818	1.4165	161.7737
C20-R50-CSS	−8.6007	1.6249	0.9720	1.6249	198.9521
C20-R100-CSS	−7.3633	1.4277	0.9727	1.4277	173.7220
C20-R50-CSA	−9.6590	1.8594	0.9599	1.8594	180.3116
C20-R100-CSA	−7.6489	1.4773	0.9894	1.4773	177.2607
C30-R0	−11.4411	2.1321	0.9835	2.1321	214.0304
C30-R50-UT	−9.2352	1.7192	0.9646	1.7192	215.2501
C30-R100-UT	−8.0791	1.5245	0.9726	1.5245	200.2383
C30-R50-CSS	−8.9869	1.6395	0.8913	1.6395	240.2039
C30-R100-CSS	−8.2890	1.5520	0.9660	1.5520	208.6901
C30-R50-CSA	−10.9689	2.0648	0.9790	2.0648	202.8224
C30-R100-CSA	−9.1777	1.6923	0.8644	1.6923	226.6056

## Data Availability

The original contributions presented in this study are included in the article. Further inquiries can be directed to the corresponding author.
